# The Embryology of the Dental Pulp

**Published:** 1902-09

**Authors:** R. R. Andrews

**Affiliations:** Cambridge, Mass.


### International Dental Journal.

Vol. XXIII. September, 1902. No. 9.

### Original Communications.[1]

### THE EMBRYOLOGY OF THE DENTAL PULP.[2]

BY R. R. ANDREWS, A.M., D.D.S., CAMBRIDGE, MASS.

At the invitation of the secretary of our Section to present a paper on some subject connected with the dental pulp, I shall consider at this time the dental pulp in its embryological aspect. Such an aspect appeals to me the more strongly from the fact that I have given special attention in earlier research work to dental embryology. In a general way I shall consider the growth of the dentine germ from the earliest signs of its development, the formation of the dentine from the germ, and lastly the fully formed and functionally mature pulp. This subject may not offer anything that is particularly new, but there are several points that I have recently been trying to clear up, and a discussion of them may prove of general interest to the Section on Stomatology.

At about the end of the second and the beginning of the third month of intra-uterine life, in the embryonic tissue of the jaw, we

[1] The editor and publishers are not responsible for the views of authors of papers published in this department, nor for any claim to novelty, or otherwise, that may be made by them. No papers will be received for this department that have appeared in any other journal published in the country.

[2] Read before the Section on Stomatology of the American Medical Association, at Saratoga Springs, June, 1902.

shall find the primary specialization of cells which are to form the dentine germ, and from which come the cells which afterwards form the dental pulp. It is in no special zone or layer of this connective tissue that the dental germ is formed, but the formation seems wholly influenced by the contact with an enamel-organ. In the presence of this organ the connective tissue cells become stimulated and active. It would appear as though they offered a resistance to its further growth, and from this resistance the enamel-organ was made to expand, thus becoming flattened and broadened. The stimulation and activity of the cells is shown by their rapid growth, which clouds the part at this point, becoming a dense focus of new growth. The tissue is seen to be actively building itself up, and this results in the formation of a papilla, around which the enamel-organ is growing like a cap or helmet. This process of new growth is a beautiful illustration of anabolic metabolism. The papilla grows to a cusp or cusps, and now becomes the dentine germ. At the end of the third and at the beginning of the fourth month the dentine germ is rather a homogeneous structure. Round cells are very numerous; they have relatively large nuclei and nucleoli. As the germ assumes the cusp shape, multiplication of cells takes place around the blood-vessels, which have grown into the base of the germ, and a jelly-like layer has formed around its outer surface. It will be found that the dental germ will grow into the depressions of the enamel-organ of a bicuspid or molar tooth, and these growths will become the dentine cusps. We also notice that the different layers of the enamel-organ are now formed, and that the sacullus is forming its layers about both enamel-organ and dentine germ. When this process is completed, these are enclosed in a sac, and thus become a dental follicle. Within the area of the dentine germ are contained all of the cells which shall develop later into the mature dentine and into the pulp of the fully formed tooth.

The round cells around the rim of the dentine papilla appear to be in a protoplasmic substance, sometimes called a zone of amorphous material. It is a hyaline structure on the outermost surface of the germ. The cells just within become richer in protoplasm, and many processes are seen to be forming from them. They are becoming branched cells. A little later the cells at the surface grow larger and assume a columnar shape, which may be caused by mechanical compression. We also see just within this layer of cells some that are pear-shaped, conical, cylindrical, and spindle-shaped. There are some authorities who have spoken of what they call elementary cells on the outer layers, and from which, they say, the odontoblasts are formed, but I have never observed anything but globular masses that are not cells, and which are found in the protoplasmic substance of the rim spoken of above. At the beginning of the fifth month these cells on the surface are seen to be undergoing a histological differentiation, as stated above, and are becoming specialized or formative cells, the odontoblasts. They are membraneless, and little more than masses of protoplasm, which are seen to be filled with great numbers of bright glistening globules of different sizes. The so-called "conjugation cells" of the German authorities are what I believe to be the pear-shaped fibre-forming cells. These are seen to be sending their processes into the intercellular spaces of the odontoblasts, and thus I believe the fibre to be formed. At this time dentinification is about to begin. How does this process of calcification take place? This we do not wholly understand, nor do we understand the chemical or physical properties of the building-materials. At this time the blood-supply is evident, and at the seventh month there is a perfect vascular system consisting of arteries, veins, and capillary net-work.

As I have said, the details of the vascular mechanism by which the odontoblasts are supplied with lime necessary to form calcified structure have not, as yet, been clearly worked out. Capillaries near the formative cells do not communicate directly with the cells, and must therefore pass the lime through the intracellular substance. The inorganic calcium which is necessary manifestly cannot be supplied as such by the organic formative cells, but must make its initial entrance into the body from without. This entrance in the fœtal state must necessarily be through the maternal circulation, and after birth it must come from the food which passes through the alimentary canal. From here it must be carried to the specialized formative cells which superintend the process of dentinification, and there is but one such distributor,—the blood-supply.

After the absorption of food into the circulation by the intestinal epithelium, chemical analysis of the blood shows the presence of two calcium salts,—the insoluble phosphate ($Ca_3(PO_4)_2$) and the soluble carbonate ($CaH_2(CO_3)_2$). It can be readily understood that the soluble carbonate can be absorbed, but how the insoluble phosphate can be absorbed is still a mooted question. It is believed, however, that it is absorbed in that same loose chemical combination with proteid in which it is found before absorption in the casein of milk and the yolk of egg. Chemical analysis has shown these two foods to be very rich in calcium. The casein and caseinogen of cow's milk, according to Bunge, contains more calcium to the litre than does lime-water. Caseinogen, according to Soldner, contains 1.55 to 2.36 per cent. of calcium. The proportion of calcium in combination with the proteid of egg-yolk has been found to be about the same.

The loose calcium-proteid combination, arriving, during its passage through the dental pulp capillaries, within the radius of the special physiological motive force of the odontoblasts, is acted upon by this vital force, and thus becomes ingested by the cells. We believe that it here becomes modified by the cytoplasm of the cell, by a chemical combination with its organic substance, and in this way calcospherites are formed. Within the cells, these globules seem to have the property of coalescing, and as they are placed by the cell against the surface to become calcified, they are found to be in many cases large globular or irregular-shaped masses. These masses, merging with others, smooth out and form the layer always found between organic and calcified tissues, where the process of calcification is taking place. This is the layer known to investigators as border-land tissue. Hoppe-Seyler asserts that the lime which hardens bone, dentine, and enamel is a double salt of carbonate and phosphate of calcium, having the formula $Ca_{10}(CO_3)_3$-$(PO_4)_6$, one equivalent of calcium carbonate, with three equivalents of calcium phosphate.

The various processes of dentinification have been demonstrated to me by many hundreds of sections cut from developing teeth at a time when calcification was beginning, and from tissue prepared as near the life of the animal as it could be, and prepared with the least possible manipulation consistent with perfect specimens.

The formation of dentine from the dentine germ proceeds substantially in the following manner: We notice that the hyaline substance on the rim of the germ, which is a protoplasm basis substance that surrounds the outer ends of the formative cells, is filling up with glistening, irregular-shaped masses that appear semisolid, many of them being globular, but all tending to form a layer of substance which is involving a portion of the outer ends of the odontoblasts. We notice that these cells themselves are filling with bright, but minute, globular bodies which are the calcospherites, that seem to have their origin within the cytoplasm of the cell; these grow larger, probably by merging with others, as they are conveyed to the calcifying surface of the layer of the rim. Mr. Mummery, of London, has described a net-work of connective-tissue fibres which were seen in bundles between the odontoblasts, and even enveloping them and passing out from them, forming a net-work just in advance of the main line of calcification. This net-work of fibres, the fibres of Mummery, serve, during the formation of the layers of dentine matrix, as a scaffolding, among which the gelatinous tissue and the calcospherites are deposited. I have described a similar net-work in developing enamel in a paper read in Berlin in 1890. In this way the calcifying layers are formed, until the dentine is completely calcified. This process is not continuous, but occurs in laminæ, as indicated by the contour lines seen in the forming specimens that have been stained. The layer which is forming is a new product, in which the lime is held in some sort of a chemical combination. In this condition it is known to be calcoglobulin, and a further chemical change forms it into the fully calcified structure. Thus the dentine is formed, layer by layer and stage by stage. We cut our sections, if we are studying the forming dentine, at a period of growth covering one of these stages, and we do not always get the same picture. Sometimes our section will show the globular formation stage, sometimes in the stage that shows the continuous band of calcoglobulin, and sometimes, though rarely, we get a picture that shows no appreciable layer between the odontoblasts and the calcified dentine. Sudduth has stated that the thickening of the dentinal wall is accomplished by a single layer of odontoblasts which begin the process, and these same cells persist throughout the life of the pulp. I cannot, with my present knowledge, agree with this statement, for I have seen earlier layers of odontoblasts being apparently used up, or engulfed within the layer forming, and other formative cells developing from the cells of the pulp-tissue just within. Oblique sections of forming dentine, and of the layer of border-land tissue, also show parts of the formative cells which become fused with it. Dr. Frank Abbott makes the statement that he has seen from time to time dentine-forming cells replaced by others, which, he says, are seen to be forming at their inner side. The layer of calcoglobulin has been called collagen; I do not believe that it is collagen. It was also formerly known as the "membrana preformativa," but this is not a membrane. The layer of odontoblasts was also known as a membrane, the "membrana eboris;" neither is this a membrane.

Morgenstein calls the layer of border-land tissue "dentinogenous substance," and thinks that it is produced by the odontoblasts giving up part of their substance; that a segmentation of the odontoblasts has taken place, somewhat as the enamel-rod is formed into segments.

There appears to be a lack of knowledge about the dental fibre, its canal, and the so-called sheaths of Neumann. We speak of the dentine tubes, or of the dentine tubuli. A tube is any long and hollow cylinder,—a pipe; tube or tubulus is certainly a misnomer. We should speak of it as the dentinal canal or dentinal canaliculus, for a canal is a duct in a body for the passage of fluids, a duct through which anything may be conducted. If we examine the cross-section of the developing tooth again, where only a narrow layer of dentine has been formed, we see on the edge of the fully calcified layer, between it and the formative cells, the transparent, hyaline layer already spoken of. It is somewhat irregular, as if it were formed by the merging of globular masses, a transitional tissue, mind you, which a further stage in the hardening process will completely calcify. It then becomes matrix or basis substance. It is formed by microscopic globules, calcospherites, within the odontoblasts. These cells appear to superintend the laying of the globules which are arranged in the substance of the gelatinous tissue, a layer of which has been formed by the pulp to receive them; they are deposited against the fully calcified matrix within the fibres of Mummery. This is the hyaline layer already spoken of. It is a layer of border-land tissue that is singularly indestructible in acids or in caustic alkalies. I have already stated that there appear to be two kinds of cells concerned in the formation of dentine,—one, a fibre-forming cell, with a long process running into the canals; the other, a matrix-forming cell, the true odontoblast. This is usually square and abrupt against the dentine, and the process which it appears to have, in many cases, I have found, belongs to the fibre-cells deeper within the pulp-tissue. As the dentine layer forms, the fibre of the fibre-cell lengthens, and against the surface or sides of this lengthening fibre the same hyaline layer is left uncalcified, as is found against the forming matrix next the formative pulp.

We frequently see two fibre-cells merged into one, caused by the lessening circumference of the forming dentine; they have merged together, one losing its identity completely at that point; and so it is with the odontoblasts. It appears to me clear that all the branching of the canaliculi must be from the merging of these fibre-cells, thus forming branches of the main fibre. The so-called sheath, then, is a transition tissue, probably the same as the tissue which remains uncalcified in the interglobular spaces in dentine. It is in no sense a separate tissue, and sheaths can only be demonstrated after full decalcification, when acids have completely destroyed the matrix. In cross-section of the canals in dentine this border-land tissue can be stained by a preparation of nitrate of silver. It acts precisely the same as it does on the hyaline layer of forming dentine,—it stains it black. Both tissues are matrix tissues in a partial state of calcification, and full calcification will take place in this border-land tissue against the fibre as age comes on, when the dentinl canals are found to be much smaller in diameter than they are in the young tooth. We may assume, then, that the so-called sheath of Neumann is but a transitional tissue only partially calcified, which may fully calcify in the future. It lines the canals in the dentinal matrix, and is only a sheath when acids have destroyed its adjoining more fully calcified substance.

In these various processes, we have considered the calcification of the deciduous central incisor. The process begins about the fourth month, the crown is nearly formed at birth, and the tooth root fully formed at the eighteenth month. Thus far it has been my purpose to describe the various processes of dentinification taking place before and after birth, as demonstrated by research work. In describing these, it has seemed necessary to repeat descriptions in order to make the subject-matter clear. In concluding, a brief description of the germ-tissue remaining after full calcification has taken place will be given. This germ-tissue now becomes the normal pulp, which is the source of nutrition and nerve-supply to the tooth. The main mass of this organ is made up of a semigelatinous matrix thickly studded with cells which do not in themselves form a continuous tissue; that is, they are not in contact with each other. They are embedded in a jelly-like substance, in which many fine fibres are seen. In the centre of the pulp-tissue the cells are less numerous than they are near the formed dentine. The cells against the dentine are no longer square and abrupt against it; they are now oval or pear-shaped, with the pointed ends conveying a fibre to a canal in the calcified matrix.

The study of many sections of the pulp of fully formed teeth has led me to believe that the pear-shaped cells fringing the outer surface of the pulp, and having fibres running into the canals of the dentine matrix, are not cells having the same function as did the formative cells, or odontoblasts, which were square and abrupt against dentine while it was forming. There are indications that the pear-shaped fibre-cells have a membrane, and they remain pear-shaped throughout the vitality of the pulp. When the pulp is irritated by the approach of caries, or from abrasion, or from some stimulation from without, the fibre-cells do not appear to take part in the formation of secondary dentine, the dentine of repair; but new formative cells are seen to be developing from the cells of the so-called conjugation layer just within.

Weil has described an intermediary layer just within the odontoblastic layer, which consists of a large accumulation of spindle-shaped cells, somewhat different from the embryonic connective-tissue cells of the main mass, which varies in breadth according to age. This intermediary layer represents the remains of what the Germans call the conjugation-cell layer,—a layer of reserve material, which sems to be a product of the growth changes of the pulp. I doubt if there is more than a remnant of it in adult teeth. Some authors assert that the cells in the centre of the pulp degenerate, that the nucleus disappears, and that there is a partial loss of their protoplasm. This is undoubtedly the case in older pulps which no longer show the rich ramifications that the younger ones do. Lymphatic vessels have never been demonstrated with certainty in the pulp-tissue. There is a net-work of undulating fibres which run from the root to the crown, parallel to the axis of the teeth. The interspaces between these cells and fibres being filled with a protoplasmic substance, their histological nature has not been determined. It is stated that the cells of the pulp show characteristic differentiation at different times in its life. There are three kinds of cells which have their origin from the embryonic connective-tissue cells by metamorphosis. These are round cells with large nucleus and scanty protoplasm, spindle-shaped cells, and irregularly shaped cells which have branching processes that freely anastomose with the spindle-shaped cells and with themselves. The changes in the cells seem to begin at the periphery and proceed towards the centre of the pulp. At the periphery we have the pear-shaped cells, then the spindle-shaped conjugation layer of cells, then the spindle-shaped and iregularly shaped cells with their anastomosing processes, and, lastly, the connective-tissue elements in the central portion of the pulp, which seem to be scant in protoplasm. These cells are not very numerous, and are in a jelly-like matrix. The blood-vessels enter at the apex, the trunk vessels resting near the centre of the pulp. Sometimes as many as three arteries are seen to enter the apical foramen. They then divide into innumerable branches, and form an extensive net-work of capillaries near the layer of the pear-shaped cells next the formed dentine. There are numerous veins also found, and these are somewhat larger than the arteries. Black tells us that the blood-vessels of the pulp are remarkable for the thinness of their walls, and that the smaller veins seem to be nothing more than endothelial cells which are placed edge on edge, or margin on margin. The arteries have a circular and longitudinal layer of muscular fibres, but these are very thinly distributed. The capillary net-work is so rich near the pear-shaped cells in the forming tooth that, when they are injected and shown under the microscope, there seems to be little room for any other tissue. The nerves of the pulp are many, the fibres being medullated and non-medullated, which enter the pulp through the apical foramen in bundles of various sizes. As they pass into the pulp they break into branches and form a rich net-work, a delicate plexus of fine nerve-filaments next the outer pear-shaped cells. It is not known just how these communicate with the fibril. It has been asserted that the finer fibres may pass between the pear-shaped cells, and wind themselves around the dentinal fibrils, passing thus into the canal. Sudduth inclines to the view that the terminal fibres unite with the pear-shaped fibre-cells, and that sensation is thus transmited by the dentinal fibril to the terminal branches of the nerves. In form a mature pulp is shaped nearly the same as the tooth to which it belongs.

